# Direct and Indirect Transcriptional Effects of Abiotic Stress in *Zea mays* Plants Defective in RNA-Directed DNA Methylation

**DOI:** 10.3389/fpls.2021.694289

**Published:** 2021-08-19

**Authors:** Thelma F. Madzima, Stefania Vendramin, Jason S. Lynn, Phebe Lemert, Katherine C. Lu, Karen M. McGinnis

**Affiliations:** ^1^Division of Biological Sciences, School of STEM, University of Washington Bothell, Bothell, WA, United States; ^2^Department of Biological Science, Florida State University, Tallahassee, FL, United States

**Keywords:** abiotic stress, RNA-directed DNA methylation, mop1, abscisic acid, *Zea mays*, epigenetics, drought

## Abstract

Plants respond to abiotic stress stimuli, such as water deprivation, through a hierarchical cascade that includes detection and signaling to mediate transcriptional and physiological changes. The phytohormone abscisic acid (ABA) is well-characterized for its regulatory role in these processes in response to specific environmental cues. ABA-mediated changes in gene expression have been demonstrated to be temporally-dependent, however, the genome-wide timing of these responses are not well-characterized in the agronomically important crop plant *Zea mays* (maize). ABA-mediated responses are synergistic with other regulatory mechanisms, including the plant-specific RNA-directed DNA methylation (RdDM) epigenetic pathway. Our prior work demonstrated that after relatively long-term ABA induction (8 h), maize plants homozygous for the *mop1-1* mutation, defective in a component of the RdDM pathway, exhibit enhanced transcriptional sensitivity to the phytohormone. At this time-point, many hierarchically positioned transcription factors are differentially expressed resulting in primary (direct) and secondary (indirect) transcriptional outcomes. To identify more immediate and direct MOP1-dependent responses to ABA, we conducted a transcriptomic analysis using *mop1-1* mutant and wild type plants treated with ABA for 1 h. One h of ABA treatment was sufficient to induce unique categories of differentially expressed genes (DEGs) in *mop1-1*. A comparative analysis between the two time-points revealed that distinct epigenetically-regulated changes in gene expression occur within the early stages of ABA induction, and that these changes are predicted to influence less immediate, indirect transcriptional responses. Homology with MOP1-dependent siRNAs and a gene regulatory network (GRN) were used to identify putative immediate and indirect targets, respectively. By manipulating two key regulatory networks in a temporal dependent manner, we identified genes and biological processes regulated by RdDM and ABA-mediated stress responses. Consistent with mis-regulation of gene expression, *mop1-1* homozygous plants are compromised in their ability to recover from water deprivation. Collectively, these results indicate transcriptionally and physiologically relevant roles for MOP1-mediated regulation of gene expression of plant responses to environmental stress.

## Introduction

The sessile nature of plants and their adaptation to terrestrial environments coincided with the evolution of whole plant and molecular responses to fluctuating environmental conditions (reviewed by Gupta et al., 2020). Extreme abiotic environments, including water scarcity, often lead to yield loss in agricultural crop plants across the globe (FAO, 2017). When osmotic stress is first detected, the initial and immediate whole plant response is often the closure of stomata, which allows the plant to conserve water within its tissues, while limiting the energy and resources expended in biological processes such as photosynthesis. More prolonged drought conditions result in responses that often limit plant growth, and are associated with developmental defects in reproductive organs, thus decreasing yield. Indeed, it has long been documented that *Zea mays* (maize) plants that experience drought stress exhibit reduced yield and the overall effects depend on the specific developmental stage at the time that stress is experienced (Claassen and Shaw, 1970). From a molecular perspective, recent studies demonstrate that whole plant responses are related to the disruption of gene regulatory networks and concomitant changes to stress-response transcriptional programs (Van den Broeck et al., 2017).

Changes in transcription at stress-responsive loci are often associated with genome-wide structural changes to chromatin that affect gene expression and can be detected as alterations in chromatin accessibility (Kim et al., 2015, reviewed by Chang et al., 2020). These changes are strongly influenced by the plant phytohormone abscisic acid (ABA), an important signaling molecule that is responsible for many processes throughout the life cycle of plants such as regulating several important stages of development, including seed germination, ABA synthesis, and signaling, and serves as a critical step in plant response to specific abiotic stress stimuli (reviewed by Ma et al., 2018, and Takahashi et al., 2020). In response to ABA, activation by phosphorylation of *trans*-acting factors initiates broad scale changes in gene expression, creating a hierarchical response that includes a combination of primary, secondary, and later stage *cis* and *trans*-acting responses at the molecular level (reviewed by Takahashi et al., 2018). It has also been observed that certain transcriptional changes in maize in response to and throughout recovery from drought stress is associated with differential enrichment for specific histone modifications (Forestan et al., 2020), and that differential DNA methylation is associated with water stress response in ABA-deficient maize mutants (Sallam and Moussa, 2021), further suggesting the overlapping regulation of ABA signaling and chromatin-mediated gene expression changes in plant stress responses. The coordinated effect of this multi-dimensional response can create whole-plant responses that originate from a molecular signal triggered by an environmental or developmental cue.

Activated *trans*-acting factors differentially regulate target chromosomal sequences, depending in part on the structure of chromatin at *cis*-regulatory elements (reviewed by Wang and Qiao, 2020). For example, evidence suggests that transcription factor binding is influenced by DNA (cytosine) methylation, although these mechanisms are not completely understood for a broad range of transcription factors (reviewed by Heberle and Bardet, 2019). Our recent investigations in maize seedlings indicates that genotypes defective in RNA-dependent DNA methylation (RdDM), a plant-specific epigenetic regulatory pathway, respond to exogenous ABA at the transcriptional level in a manner distinct from wild type plants (Vendramin et al., 2020). Genotype-specific changes in CHH (H = A, T or C) methylation were also observed at some loci transcriptionally responsive to ABA (Vendramin et al., 2020), which is consistent with prior observations for targets of RdDM (Gent et al., 2014). While this indicates that there is a relationship between transcriptional regulation by RdDM and ABA-mediated responses in maize, this association does not clearly distinguish between causality, dependence or coincidence. Interpretation is confounded by the fact that each regulatory network (ABA and RdDM) has primary and cascading indirect effects influencing to gene expression and methylation.

With regards to hormone signaling in response to environmental stress stimuli, time course experiments are a useful way to elucidate hierarchical relationships in complex regulatory networks, as the primary responses are generally expected to be triggered immediately following the stimulus, and the secondary and other downstream responses may require some time to occur. Time course analysis of ABA-regulatory networks in the model plant *Arabidopsis thaliana* suggest that ABA responsive changes in gene expression may be spread across an initial response period from 1 to 8 h after exposure to exogenously applied ABA (Song et al., 2016). To better understand the specific regulatory relationships of epigenetic gene regulation and abiotic stress responses in plants, changes in gene expression in maize plants that were either wild type or defective in RdDM were compared after 1- or 8-h exposure to exogenous ABA. Because these early responses can have long-term developmental and physiological effects on stressed plants, we also investigated the whole-plant responses of plants defective in RdDM to a severe drought simulation by withholding water for 14 days.

## Materials and Methods

### Plant Materials and Growth Conditions

Maize (*Zea mays*) plants with the *mop1-1* mutation introgressed into the B73 inbred as previously described (Madzima et al., 2014) were used for this analysis. Homozygous wildtype (*Mop1* WT) and homozygous mutant (*mop1-1*) sibling progeny resulting from the self-pollination of an ear of a heterozygous plant were used. Seedlings were genotyped as previously described (Madzima et al., 2014).

*For abscisic acid treatment of maize seedlings*: Seedlings were grown in greenhouse conditions (16 h light period, 25°C, 50% humidity) in the Department of Biological Science at Florida State University (319 Stadium Drive) until they reached the V3 stage. At the V3 stage, maize seedlings were removed from the soil, roots were rinsed in water, dried, and then submerged in a 1 L beaker with 250 mL of liquid Murashige and Skoog (MS) media (Sigma Aldrich, M6899) with 50 μM ABA [ABA; (Sigma Aldrich, (+/–) Abscisic Acid, A1049)] or without ABA (MS) for 1 h (this study) or 8 h (Vendramin et al., 2020) in greenhouse conditions. After the incubation period, roots where removed and seedlings were immediately flash frozen in liquid nitrogen and stored at −80°C until use.

*For severe drought simulation on maize plants:* Plants were grown in greenhouse climate-controlled conditions (25°C, 50% humidity) at the Florida State University Mission Road Research Facility, Tallahassee, Florida, USA in January of 2017. B73 seeds were sown alongside as a control for drought response. Healthy B73, homozygous *Mop1*, and homozygous *mop1-1* seedlings were then transplanted into 300 size pots and later into 2,000 size pots ~35 days after sowing (DAS). Plants were randomly assigned to severe drought treatment (water withheld for 14 days) or normally watered groups. B73 (11 plants), *Mop1* (11 plants), and *mop1-1* (16 plants) individuals were in the normally watered control group and *B73* (10 plants), 16 *Mop1*(16 plants), and *mop1-1* (11 plants) individuals were in the drought treated group. The non-uniformity in sample number per category was due to premature death for a few individuals. Drought-treatment began once the individual plant reached the V6 stage to control for variation in development between samples/genotypes. After 14 days, plants entered the recovery phase by application of 7.5 L of water to the soil. After recovery, plants from the drought treated group were normally watered throughout the duration of the experiment. The tip of the V8 leaf (~4 cm) was dissected from the maize plants, immediately frozen in liquid nitrogen, and stored at −80°C until use.

### Physiological Observations of Drought-Responsive Traits

All observations were made daily between the hours of 10:00 and 14:00. The growth stages of individual plants were determined using the leaf collar method (Nielsen, 2019). Plant growth was monitored beginning after seed germination (VE) and continued through the last collared leaf below the tassel (≤ V18). Daily observations were made to track the emergence of ears, tassels, silks, pollen shed, and the anthesis-silking interval (ASI) which is defined as the number of days between the first pollen shed and silk emergence (Bolanos and Edmeades, 1996). We determined the “effective tassel branch score” by inspecting each tassel and determining the ratio of functional tassel branches (branches with anthers) to total tassel branches (reported as a percentage). We determined the plant height at 90 DAS by measuring the length between the first node above the soil and the tip of the longest tassel branch. We determined the average internodal length by measuring the internodal distance of the three apical internodes above the V4 leaf node.

### Total RNA Isolation, RNA Library Preparation and RNA-Sequencing

Total RNA was extracted as previously described (Vendramin et al., 2020). Briefly, frozen tissue was finely ground into powder in liquid nitrogen and homogenized before total RNA extraction was performed using TRI reagent® according to manufacturer's instructions (Molecular Research Center, 18080-051). RNA samples were DNase treated (RQ1 RNase-Free DNase, Promega, M6101) and purified using RNA clean and concentrator^TM^ 25 (Zymo Research, R1018).

Three biological replicates were used for all RNA-seq experiments for each treatment and genotype, for a total of 12 samples per time point: 1 h (this study) and 8 h (Vendramin et al., 2020). The final sample concentration was quantified by Qubit. RNA library preparation (NEBNext^®^ Ultra™ II kit, NEB, E7760) and Illumina paired-end 150 bp (PE 150) sequencing were performed by Novogene Corporation (Sacramento, California). The 1 h samples were sequenced using the Illumina NovaSeq 6000 platform, whereas the previous reported 8 h samples (Vendramin et al., 2020) were sequenced using the Illumina HiSeq 2,500 platform. More than 20 million reads were obtained per library.

### Read Alignment, Batch Correction and Differential Gene Expression Calling

Bioinformatics analysis was performed by Linkage Analytics, LLC (Denver, CO). To ensure a consistent and re-producible computation environment, the workflow was containerized using Singularity (3.6.4) (Kurtzer and Sochat, 2017) and the data workflow steps were defined using Snakemake (5.30.1) (Koster and Rahmann, 2018) and read quality control was assessed using fastp (0.21.0) (Chen et al., 2018). Reads from the 1 h and 8 h sequencing batches were processed simultaneously. FASTQ adapters were trimmed by Cutadapt 1.8.1 (Martin, 2011) Reads were mapped to the B73 maize genome (AGP B73v4) (Jiao et al., 2017) by HISAT2 v2.2.1 (Pertea et al., 2016). Transcripts were assembled *de novo* to allow for inclusion of transcripts that are not included in the reference genome annotation and quantified using StringTie v2.1.4 (Pertea et al., 2016). Gene count matrices were generated from this data using the prepDE.py python script available in the StringTie website (http://ccb.jhu.edu/software/stringtie/index.shtml?t=manual). These matrices were used by the Bioconductor package edgeR 3.28.1 (Chen et al., 2016) in R for differential expression analysis in order to identify upregulated and downregulated genes for the four different genotypes under two treatments. Low-abundance counts of <0.58 cpm were removed using the DESeq2 filtering method (statquest.org/2017/05/16/statquest-filtering-genes-with-low-read-counts/); (Love and Huber, 2014) incorporated into the edgeR pipeline, and genes with an adjusted *p*-value of ≤ 0.05 and an absolute log_2_-fold change (FC) value of ≥ 0.95 were considered as differentially expressed for both upregulated and downregulated genes.

### Gene Ontology Analysis and Hierarchical Clustering of Significantly Enriched GO Terms and DEGs

Singular Enrichment Analysis (SEA) was performed using the web-based tool agriGO v2.0 (Tian et al., 2017) with the B73 reference version 4 (AGOv4) gene annotations to determine enriched gene ontology terms (GO complete) associated with differentially expressed genes.

Fisher's statistical test, Hochberg (FDR) multi-test adjustment method with a significance level of <0.05 and minimum number of mapping entries of 10 genes per GO-term. The GO term enrichment was generated by hierarchically clustering the log10 of the total GO term percentage of a set of genes that were upregulated or downregulated in wildtype or mutant in response to ABA.

## Results

### Early ABA Treatment Is Sufficient to Induce Unique Categories of Differently Expressed Genes in *mop1-1* Mutants

To identify genes that are immediately responsive to epigenetic regulation under abiotic stress conditions, RNA from maize seedlings exposed to 1h of abscisic acid (ABA) and nutrient solution without ABA (MS) in *mop1* wildtype (WT) and mutant (*mop1-1*) genotypes was subjected to RNA-sequencing (RNA-seq) and transcriptome analysis as previously described (Vendramin et al., 2020). An average of ~33 million 150 bp paired end raw reads were obtained per sample (Table 1) and mapped to the B73 maize genome (AGP B73v4) (Jiao et al., 2017). Significant differentially expressed genes (DEGs) between *mop1* genotypes and 1h ABA treatments were categorized into four pairwise comparisons designated “analysis groups” (1h Groups A–D; Table 2) as genes with a two-fold expression change (log_2_ FC ≥ 0.95, FDR < 0.05) and identified by making direct comparisons between genotypes and treatments (Table 2). The DEGs in the four analysis groups were further sub-divided based on gene expression patterns (up- or down-regulated; e.g., 1h A-up and 1 h A-down) (Table 2; File 1) and subjected to further analysis.

**Table 1:** Summary of RNA-seq libraries and read mapping per time-point, genotype, and treatment.

**Timepoint**	**Genotype & Treatment**	**Replicate**	**Total raw reads**	HISAT2 slope filter threshold^*^	**Mapped reads**	**% Mapped reads**	**Uniquely mapped reads**	**% Uniquely mapped reads**
1 h(this study)	*mop1-1* mutant ABA	1	32349928	−0.2	32183321	98.2	29348167	91.19
		2	23272446	−0.2	23161089	98.29	21341515	92.14
		3	33691278	−0.2	33515782	98.3	30773769	91.82
	*mop1-1* mutant control	1	35425161	−0.2	35251763	98.31	32054938	90.93
		2	36809285	−0.2	36630297	98.31	33089359	90.33
		3	33801811	−0.2	33627611	98.26	30993247	92.17
	*Mop1* WT ABA	1	32652171	−0.2	32489954	98.34	29886735	91.99
		2	37341781	−0.2	37146123	98.23	34191248	92.05
		3	30156617	−0.2	30004706	98.22	27603302	92
	*Mop1* WT control	1	31505710	−0.2	31337674	98.27	28676534	91.51
		2	36734172	−0.2	36545051	98.33	33554582	91.82
		3	35711598	−0.2	35537773	98.42	32637773	91.84
8 h Vendramin et al. (2020)	*mop1-1* mutant ABA	1	21775951	−0.6	21329876	97.3	19336347	90.65
		2	22373108	−0.6	21981634	96.91	19388351	88.2
		3	21583557	−0.6	21109587	96.84	18349596	86.93
	*mop1-1* mutant control	1	24469219	−0.6	23915909	96.38	20602662	86.15
		2	24142476	−0.6	23706188	97.65	21382476	90.2
		3	23023844	−0.6	22521807	95.33	19520082	86.67
	*Mop1* WT ABA	1	22263417	−0.6	21821952	96.02	18855244	86.4
		2	23131739	−0.6	22765701	96.49	20024820	87.96
		3	21771139	−0.6	21360341	96.87	18914163	88.55
	*Mop1* WT control	1	22307497	−0.6	21875147	96.53	18844702	86.15
		2	22220268	−0.6	21837022	95.8	18620624	85.27

* *HISAT2 filters reads based on a threshold defined by the slope a linear function between mapping quality score and read length. See Supplementary Figure 1 and the HISAT2 manual entry for “–score-min” for details*.

**Table 2:** Analysis Groups for 1 h.

**Pair-wise comparison**	**Analysis group**	**Expression pattern**	**Significant^a^ DEGs**	**2FC Significant^a^ DEGs**	**Total Significant^a^ DEGs**	**Total 2FC Significant^a^ DEGs**
Mutant ABA vs. Mutant MS at 1 h	A_UP	upregulated	11	9	97	72
	A_DOWN	downregulated	86	63		
WT ABA vs. WT MS at 1 h	B_UP	upregulated	22	21	66	61
	B_DOWN	downregulated	44	40		
Mutant ABA vs. WT ABA at 1 h	C_UP	upregulated	882	646	1,849	1,171
	C_DOWN	downregulated	967	525		
Mutant MS vs. WT MS at 1 h	D_UP	upregulated	413	395	604	552
	D_DOWN	downregulated	191	157		
Total DEGs					2,616	1,856
Number of DEGs in more than one analysis group					871 (33%)	737 (40%)
Number of DEGs in only one analysis group					1,745 (67%)	1,119 (60%)

a *Significant genes are DEGs with a p-value and FDR, 0.05*.

**Figure 1:**
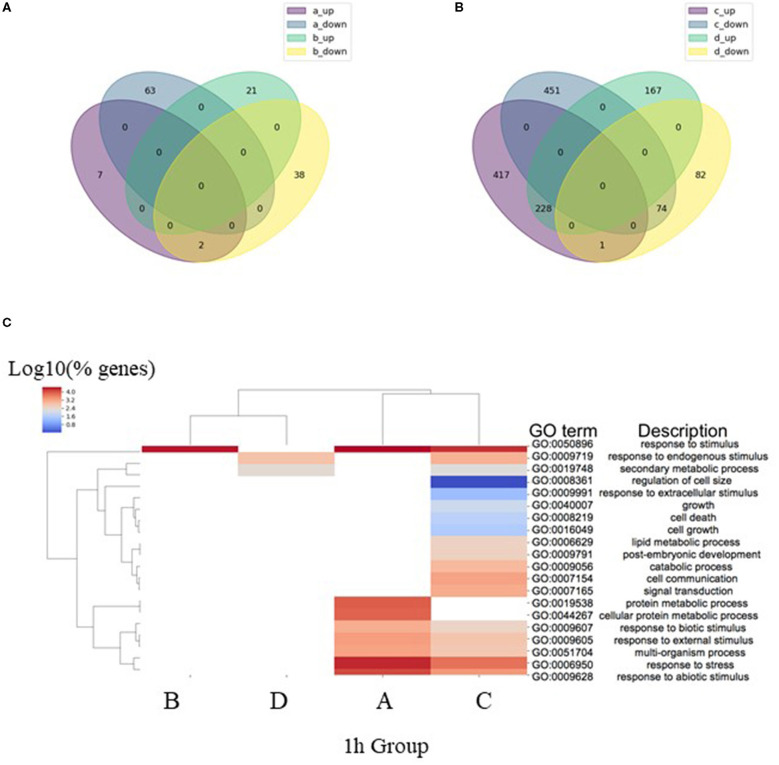
Analysis of differentially expressed genes (DEGs) after 1 h ABA treatment. **(A)** Venn diagram of overlap in the identity of DEGs (log2 FC ≥ 0.95, FDR < 0.05) after 1 h treatment with ABA compared to control in homozygous mutant (a_up and a_down) and wild type plants (b_up and b_down). **(B)** Venn diagram of overlap in the identity of DEGs (log2 FC ≥ 0.95, FDR < 0.05) in homozygous mutant compared to wild type plants after 1 h treatment with ABA (c_up and c_down) or 1 h treatment with MS (d_up and d_down). **(C)** Comparisons of enriched biological process (BP) Gene Ontology (GO) terms in DEGs were made between 1 h analysis Groups A–D (Table 2), representing DEGs identified in pairwise comparisons homozygous mutants treated with ABA or MS (Group A), wild type plants treated with ABA or MS (Group B), 1 h of ABA treatment for homozygous mutant or wild type plants (Group C) and 1 h treatment with MS for homozygous mutants or wild type plants (Group D). Heatmap illustrates hierarchical clustering of log10 (% genes) of significant GO terms enriched in each expression comparison (FDR < 0.05, minimum of 10 genes per GO term). No color (white) indicates that there was no enrichment for the GO term in the dataset.

The total number of significant DEGs with two-fold change (2FC) in expression identified in the four analysis groups after 1 h of ABA-induction (1 h Groups A–D) included 1,856 genes, where, 737 (40%) of these genes were found to be common to more than one group, resulting in 1,119 (60%) DEGs unique to an individual analysis group (Table 2; Figure 1). After 1 h of ABA induction, only ~7% of the total 2FC DEGs were differentially expressed in WT and *mop1-1* genotypes relative to their own control (1 h Groups A and B). These transcriptional responses are genotype-specific as there was also almost no overlap between the DEGs identified in each of these analysis groups (1 h Groups A and B) (Figure 1A). The majority of DEGs (63%) were identified in comparisons that included both genotype and treatment (Table 2 Group C*; mop1-1* ABA/WT ABA). A comparison between Group C DEGs with control plants of the same genotypes (*mop1-1* MS/WT MS from Group D), revealed 417 and 451 up- and down-regulated genes, respectively, that are uniquely responsive to ABA treatment and loss of MOP1 activity (Figure 1B). These 868 genes were subjected to a more in-depth analysis to identify primary and indirect MOP1 specific targets.

Gene ontology (GO) analysis was used to predict the biological processes of all annotated genes in each of the four analysis groups (1h Groups A-D; FDR < 0.05). As expected, the GO term for response to stimulus (GO:0050896) was highly enriched in all 1 h analysis groups, except for the comparison constituting a genotype control of mutant and wild type plants treated with MS (Group D; Figure 1C). The diversity of enriched DEGs was enhanced in *mop1-1* mutants subjected to ABA (1 h Groups A and C) relative to WT plants (Group B) or the genotype control (Group D) (Figure 1C). These *mop1-1* ABA unique categories include biological processes associated with cell growth and size (Figure 1C).

### In *Mop1-1* Mutants, the Most Distinct Changes in Gene Expression Occur Within the Early Stages of ABA Induction

To identify genes that respond to ABA and MOP1 in a temporal manner, the mapped reads from RNA-seq after 1 h (this study) and 8 h (Vendramin et al., 2020) of ABA induction were simultaneously, bioinformatically processed and mapped to the B73 reference genome (AGP B73v4) (Jiao et al., 2017) and used in subsequent analysis (Tables 1–3). Due to the differences in sequencing depth as a result of use of different Illumina sequencing platforms (HiSeq vs. NovaSeq) between the two timepoints, we normalized the read quality score threshold used in HISAT2 (“–score-min”) between platforms. Based on consistency between replicates as well as differences in distributions of mapping qualities between sequencing platforms, the HISAT2 “–score-min” parameter was chosen to normalize the number of uniquely mapped reads across datasets (Table 1; Supplementary Figure 1).

**Table 3:** Analysis Groups for 8 h.

**Pair-wise comparison**	**Vendramin et al. Analysis group**	**Analysis group**	**Expression pattern**	**Significant^a^ DEGs**	**2FC Significant^a^ DEGs**	**Total Significant^a^ DEGs**	**Total 2FC Significant^a^ DEGs**
Mutant ABA vs. Mutant MS at 8 h	V	A_UP	upregulated	2,229	1,100	4,924	2,550
	VI	A_DOWN	downregulated	2,695	1,450		
WT ABA vs. WT MS at 8 h	I	B_UP	upregulated	1,530	957	3,145	1,903
	II	B_DOWN	downregulated	1,615	946		
Mutant ABA vs. WT ABA at 8 h	VII	C_UP	upregulated	510	448	796	609
	VIII	C_DOWN	downregulated	286	161		
Mutant MS vs. WT MS at 8 h	III	D_UP	upregulated	354	354	458	456
	IV	D_DOWN	downregulated	104	102		
	Total DEGs					9,323	5,518
	Number of DEGs in more than one analysis group					5,820 (62%)	3,986 (72%)
	Number of DEGs in only one analysis group					3,503 (38%)	1,532 (27%)

a *Significant genes are DEGs with a p-value and FDR, 0.05*.

Predictably, the overall number of DEGs increases with increasing time. Eight h of ABA treatment resulted in more genes exhibiting differential expression, but most of the genes were detected in multiple analysis groups, resulting in a lower percentage of DEGs being unique to one analysis group at 8 h (27%) compared to 60% unique DEGs observed after 1 h of ABA-induction (Tables 2, 3). Consistently, while there was almost no overlap between wildtype and mutants DEGs in 1 h Groups A and B (Figure 1), there was more overlap between Groups A and B after 8 h of ABA treatment (Supplementary Figure 2; Supplementary Table 2). For analysis groups C, there were more significant DEGs at 1 h of ABA treatment, compared to the same comparison after 8 h (Tables 2, 3). This suggests that the most distinct changes in gene expression between these genotypes occurs within the early stages of ABA induction.

**Figure 2:**
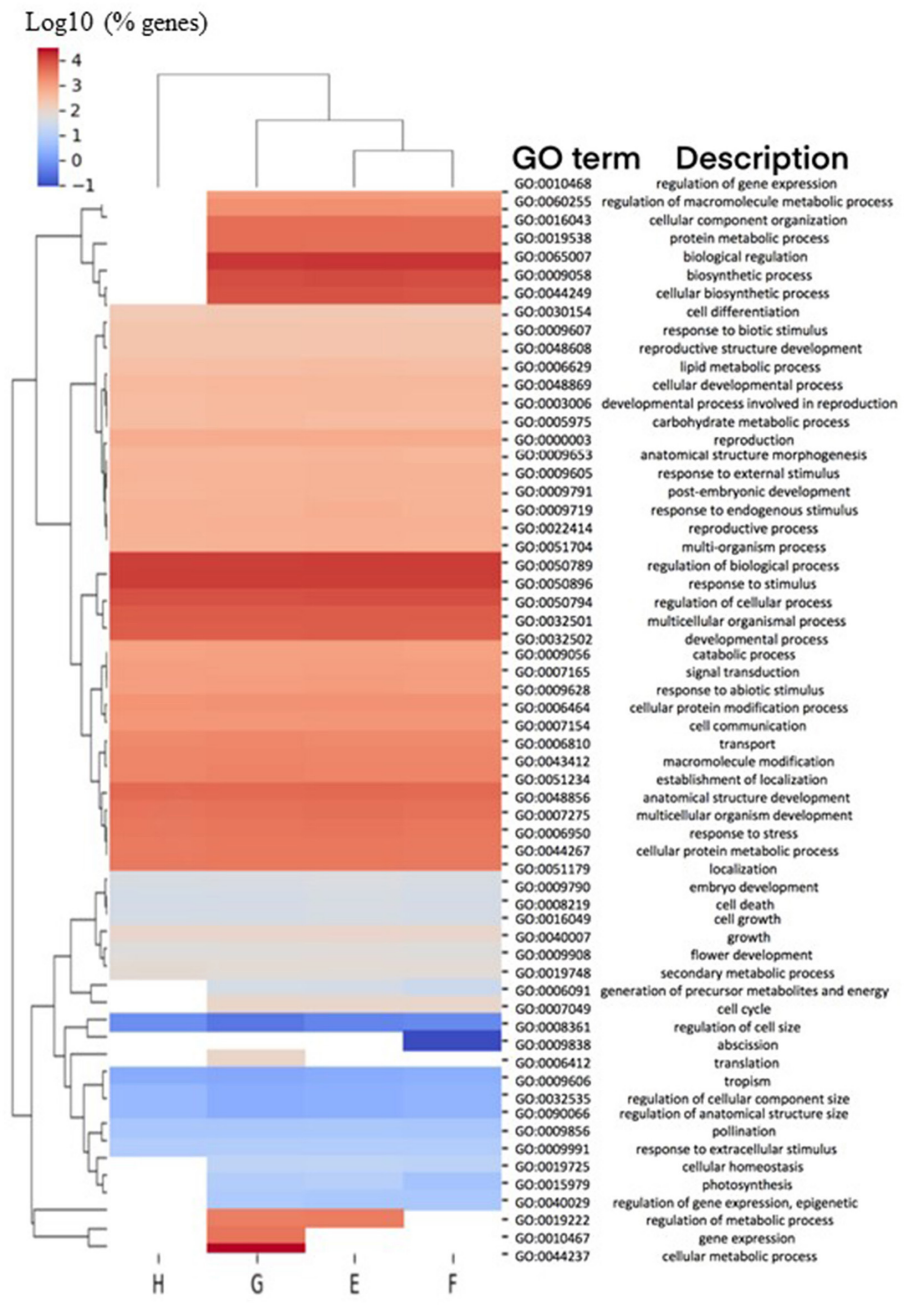
Biological processes associated with DEGs in 8 h/1 h comparisons. Comparisons of enriched biological process (BP) Gene Ontology (GO) terms in DEGs were made between 8 h/1 h analysis Groups E-H (Table 4), representing DEGs from pairwise comparisons between homozygous mutant plants treated with ABA for 8 h or 1 h (Group E), homozygous mutant plants treated with MS for 8 h or 1 h (Group F), wild type plants treated with ABA for 8 h or 1 h (Group G), and wild type plants treated with MS for 8 h or 1 h (Group H). Heatmap illustrates hierarchical clustering of log10 (% genes) of significant GO terms enriched in each expression comparison (FDR < 0.05, minimum of 10 genes per GO term). No color (white) indicates that there was no enrichment for the GO term in the dataset.

Genes from the 8 h and 1 h samples were directly compared with each other (8 h/1 h) and categorized into four different pairwise comparisons, designated Groups E–H (Table 4). The total number of significant 2FC DEGs identified in these four 8 h/1 h analysis groups was 34,147 genes, representative of the magnitude of changes in gene expression that occur over time. However, 29,610 (87%) genes were found to be common to more than one analysis group, where only 4,537 (13%) were found to be unique to an individual group (Table 4; Supplementary Figure 3). This observation is consistent with the similarities in the enriched GO terms per group (Figure 2), with the least diverse biological processes observed in the wildtype control group (Group H). GO terms associated with biological regulation (GO:0065007), regulation of biological process (GO:0050789) and response to stimulus (GO:0050896) were commonly highly represented terms across all groups (Figure 2).

**Table 4:** Analysis Groups for 8 h vs. 1 h.

**Pair-wise comparison**	**Analysis group**	**Expression pattern**	**Significant^a^ DEGs**	**2FC Significant^a^ DEGs**	**Total Significant^a^ DEGs**	**Total 2FC Significant^a^ DEGs**
Mutant ABA 8 h vs. Mutant ABA 1 h	E_UP	upregulated	7,201	5,483	13,381	7,989
	E_DOWN	downregulated	6,180	2,506		
Mutant MS 8 h vs. Mutant MS 1 h	F_UP	upregulated	7,401	5,726	14,587	9,133
	F_DOWN	downregulated	7,186	3,407		
WT ABA 8 h vs. WT ABA 1 h	G_UP	upregulated	8,188	5,674	16,306	8,621
	G_DOWN	downregulated	8,118	2,947		
WT MS 8 h vs. WT MS 1 h	H_UP	upregulated	6,039	5,273	10,674	8,404
	H_DOWN	downregulated	4,635	3,131		
Total DEGs					54,948	34,147
Number of DEGs in more than one analysis group					49,465 (90%)	29,610 (87%)
Number of DEGs in only one analysis group					5,483 (10%)	4,537 (13%)

a *Significant genes are DEGs with a p-value and FDR, 0.05*.

### MOP1-Dependent siRNAs and Gene Regulatory Networks (GRNs) Predict Immediate and Indirect Responses to Abiotic Stress

**Figure 3:**
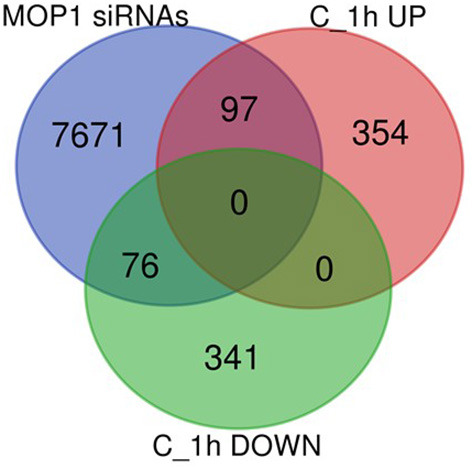
Identification of unique Group C genes with homology with MOP1-dependent siRNAs. Venn diagram of overlap between the identities of 451 upregulated (C_1h UP) and 417 downregulated (C_1h DOWN) genes in homozygous mutant plants treated for 1 h with ABA versus wild compared with genes with homology with MOP1-dependent 24nt siRNAs within their promoters (MOP1 siRNAs; Vendramin et al., 2020).

To distinguish between primary and indirect targets of epigenetic regulation under abiotic stress conditions, the 868 genes (451 up- and 417 down-regulated) identified to be uniquely associated with ABA treatment and loss of MOP1 activity (Group C; Figure 1B) were further analyzed for a specific connection with MOP1-mediated RdDM. MOP1 is required for the production of the majority of siRNAs at loci undergoing RdDM (Gent et al., 2014), therefore these genes were compared with a list of genes having promoter homology with MOP1-dependent siRNAs (Vendramin et al., 2020). This comparison identified 97 up- and 76 down-regulated genes from 1 h Group C that are predicted to be direct MOP1-regulatory targets based on homology with siRNAs (Figure 3; Supplementary Table 3), suggesting that MOP1-mediated RdDM is involved in early responses to ABA at these specific genes. It is plausible that these 173 genes are primary targets of MOP1 that in turn influence downstream gene expression in response to ABA. Because these genes are differentially responsive in *mop1-1* plants very early after ABA treatment, these genes were designated as MOP1-dependent immediate responsive genes (MIMs). Gene ontology analysis of these genes revealed that there were more significant (FDR < 0.05) enriched GO terms associated with the 97 up-regulated genes compared with the 76 down-regulated genes (Table 5). This suggests that in response to ABA, MOP1-dependent activity and siRNAs are directly associated with regulation of specific biological processes, whereas the siRNAs associated with downregulated genes (MOP1-independent) may be indirect, not RdDM targets and/or have less specific biological roles in relation to ABA responses.

**Table 5:** GO terms for biological processes associated with Group C 1 h genes with homology with MOP1 dependent siRNAs.

**GO Accession**	**GO term**	**Number of genes**	**Query total**	***p*-value**	**FDR**
**Group C_1h_upregulated**					
GO:0009628	response to abiotic stimulus	34	97	3.50E-07	8.50E-06
GO:0009719	response to endogenous stimulus	30	97	2.00E-07	8.50E-06
GO:0050896	response to stimulus	78	97	2.00E-06	3.30E-05
GO:0006950	response to stress	50	97	1.30E-05	0.00016
GO:0019222	regulation of metabolic process	44	97	0.0014	0.014
GO:0007154	cell communication	32	97	0.0021	0.017
GO:0007165	signal transduction	29	97	0.0025	0.018
GO:0050789	regulation of biological process	71	97	0.0036	0.022
GO:0010468	regulation of gene expression	30	97	0.0068	0.037
GO:0009791	post-embryonic development	19	97	0.0076	0.037
GO:0060255	regulation of macromolecule metabolic process	34	97	0.0086	0.038
GO:0050794	regulation of cellular process	60	97	0.011	0.046
**Group C_1h_downregulated**					
GO:0019748	secondary metabolic process	16	73	8.20E-08	3.60E-06

To understand how MIMs potentially influence downstream transcriptional responses to 1 h ABA treatment in maize, a gene regulatory network (GRN) (Huang et al., 2018) was used to predict targets of these 97 and 76 up- and down-regulated genes, respectively. Twenty one of the 97 (~22%) upregulated genes and 5 of the 76 (~7%) downregulated genes had predicted regulatory targets based on the GRN, and the majority of these 26 genes are transcription factors implicated in drought, ABA and stress responses based on homology and phenotypic characterization in other studies (Table 6). The predicted GRN targets of the 21 Group C 1 h upregulated MIMs included a total of 16,748 genes and the predicted GRN targets of the 5 Group C 1 h down-regulated MIMs included a total of 4,221 genes across all tissues types and datasets in the GRN (Table 6; File 3). Some of these genes (~14%) were duplicated in the two lists of targets predicted by the GRNs and overall there were 18,014 unique target genes. These targets predicted by the GRN could be considered indirect (secondary or more downstream) targets of MOP1 responsive factors, because they are predicted to be regulated by genes with evidence of direct MOP1-mediated regulation. This group of genes were collectively designated as MOP1-dependent indirectly responsive genes (MINs).

**Table 6:** 1 h Group C Genes with predicted regulatory targets based on a gene regulatory network.

**Gene ID**	**Annotation**	**DE C_1h**	**Number of GRN predicted targets**			
			**Leaf**	**Root**	**SAM**	**Seed**
Zm00001d047999	bHLH TF^*^ 9^a^	Down	334	6	242	153
Zm00001d049173	WRKY TF 36^a^	Down	1318	1630	0	0
Zm00001d003293	NAC TF 111^a^	Down	0	49	0	743
Zm00001d017084	NAC TF 13^a^	Down	110	37	90	120
Zm00001d031728	AP2-EREBP TF 79^a^	Down	0	616	0	0
Zm00001d051239	AP2-EREBP TF 170^a^	Up	122	833	355	0
Zm00001d002025	AP2-EREBP TF 24^a^	Up	3051	293	217	1483
Zm00001d002364	AP2-EREBP TF 97^a^	Up	610	742	551	167
Zm00001d002867	AP2-EREBP TF 154^a^	Up	140	9	131	0
Zm00001d004358	ABI3-VP1 TF 28^a^	Up	109	0	145	941
Zm00001d005609	protein phosphatase 2C A5^b^	Up	486	963	507	202
Zm00001d006169	DREB 1A^c^	Up	0	31	795	0
Zm00001d011589	NAC TF 134^a^	Up	71	136	374	121
Zm00001d012285	MYB-related TF 55^a^	Up	66	1327	402	603
Zm00001d014938	trihelix TF 22^a^	Up	1700	58	784	1603
Zm00001d015521	G2-like TF 24^a^	Up	334	35	91	1679
Zm00001d017422	Homeobox TF 41^a^	Up	104	432	1167	585
Zm00001d018119	bHLH TF 161^a^	Up	1033	336	174	485
Zm00001d018178	bZIP TF 4^a^	Up	558	805	304	174
Zm00001d024200	C2C2 CO-like TF 19^d^	Up	1388	1095	740	14
Zm00001d025055	protein phosphatase 2C A9^b^	Up	20	65	1911	734
Zm00001d027901	ZIM TF 16^a^	Up	126	1221	859	136
Zm00001d028752	protein phosphatase 2C 26^e^	Up	114	231	835	91
Zm00001d041491	CCAAT-HAP2-TF 212^a^	Up	62	106	801	472
Zm00001d047732	protein phosphatase 2C 32^f^	Up	33	182	269	67
Zm00001d050195	WRKY TF 94^a^	Up	145	1060	142	198

* *TF = Transcription factor*.

a *Yilmaz et al. (2009)*.

b *Xiang et al. (2017)*.

c *Qin et al. (2004)*.

d *Song et al. (2018)*.

e *Lu et al. (2020)*.

f *NCBI (https://www.ncbi.nlm.nih.gov)*.

**Figure 4:**
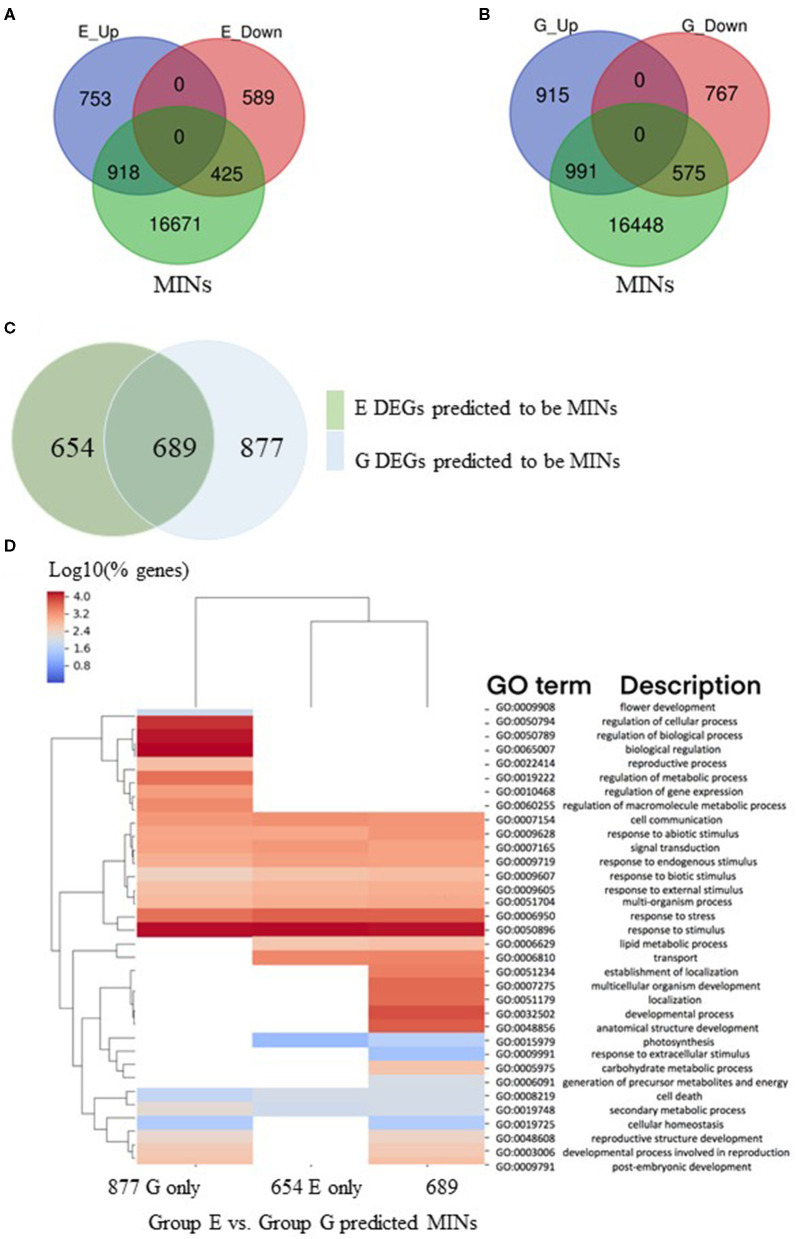
Differentially expressed genes predicted to be downstream regulatory targets of MOP1. A gene regulatory network was used to predict the regulatory targets of genes designated as MOP1-dependent immediate responsive genes (MIMs). The predicted regulatory targets of MIMs were designated as as MOP1-dependent indirectly responsive genes (MINs). **(A)** Gene identities of MINs were compared to those of DEGs identified in a comparison between homozygous *mop1-1* plants treated with ABA for 1 h vs. 8 h (E_Up and E_Down), venn diagram illustrates overlap between these groups. **(B)** Gene identities of MINs were compared to those of DEGs identified in a comparison between wild type plants treated with ABA for 1 h vs. 8 h (E_Up and E_Down), venn diagram illustrates overlap between these groups. **(C)** The identity of MINs that were differentially expressed in homozygous *mop1-1* plants treated with ABA for 1 h vs. 8 h (E DEGs) compared to wild type plants treated with ABA for 1 h vs. 8 h (G DEGs) were compared, venn diagram illustrates the overlap between these groups. **(D)** Comparisons of enriched biological process (BP) Gene Ontology (GO) terms in DEGs were made between MINs that were identified as differentially expressed in homozygous *mop1-1* plants treated with ABA for 1 h vs. 8 h (Group E) compared to wild type plants treated with ABA for 1 h vs. 8 h (Group G) or in both analysis groups (E + G). Heatmap illustrates hierarchical clustering of log10 (% genes) of significant GO terms enriched in each expression comparison (FDR < 0.05, minimum of 10 genes per GO term). No color (white) indicates that there was no enrichment for the GO term in the dataset.

The 18,014 MINs (Table 6; File 3) were compared with genes in analysis groups E and G (Table 4), representing genes differentially expressed in a temporal manner (8 h/1 h) after treatment with ABA in mutant and wild type, respectively (Figure 4A). This analysis revealed that in the mutant genotype, 54% of upregulated and 42% of downregulated genes with expression changes from 1 h to 8 h in the presence of ABA were identified as MINs (Figure 4A). A similar comparison in wild type identified that 52% of upregulated and 42% of downregulated were identified as MINs (Figure 4B). This initial observation suggests that over time, there is a similar magnitude of indirect effects in MOP1-regulated targets between mutant and wild type plants subjected to abiotic stress stimuli. However, further analysis revealed qualitative and functional differences in DEGs, as the identity of genes did not completely overlap (Figure 4C). Specifically, there were 689 (44% from wildtype (G) and 51% from mutant) putative indirect DEGs between 1 h and 8 h of ABA treatment common to both genotypes (Figure 4C; Supplementary Table 4). A gene ontology analysis was used to identify genes that fall into the three categories (Figure 4D) and GO terms associated with response to stimulus were conserved in these three categories. It appears that *mop1-1* mutants are not expressing some of the developmental genes required for MOP1-mediated responses to ABA. This comparison of DEGs between mutant and wild type may be indicative of the role of MOP1 in maize development in response to some abiotic stress stimuli. This association is also supported by recent work in other labs, indicating a role for RdDM or other chromatin-mediated regulatory events in stress response in maize (Forestan et al., 2016, Forestan et al., 2017, Forestan et al., 2020).

### MOP1 Is Required for Recovery From Drought Stress

**Figure 5:**
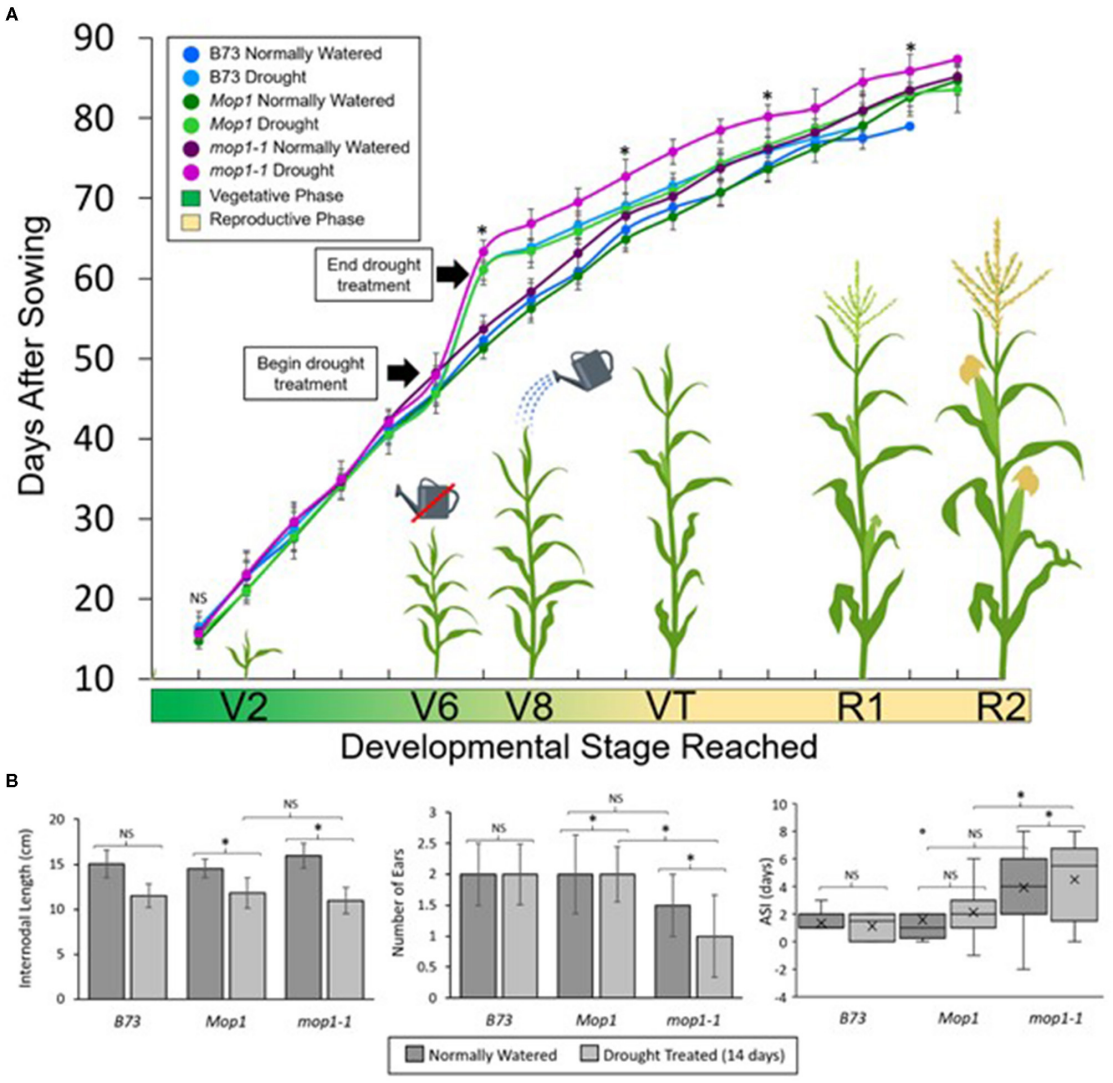
Effects of drought treatment on growth rate and reproductive development. **(A)** The gradient on the x-axis represents the approximate vegetative to reproductive developmental transitions, from the growth stage where two vegetative leaves have emerged (V2) through the 8 vegetative leaf stage (V8), and the reproductive stages where tassels and ears are present (VT) through later stages of flowering/seed set (R1 to R2). Individual points indicate mean days after sowing (DAS) when the developmental stage was reached for the indicated genotypes and treatment groups, and error bars show standard deviation within groups. Data is shown for B73 inbred lines under different watering conditions, and wild type (*Mop1*) and homozygous *mop1-1* individuals segregating within a family. Significant differences (*p* < 0.05) between drought-treated *mop1-1* and *Mop1* plants are indicated for selected points with an asterisk (*) or NS for no significance. **(B)** Physiological observations from drought stress experiment showing average internodal length, number of ears per plant, and anthesis-silking interval (ASI). Significant differences (*p* < 0.05) between groups are indicated by an asterisk (*) or no significance (NS).

To determine the role of MOP1 in drought stress response and recovery at the whole-plant level, we characterized the vegetative and reproductive developmental consequences of a severe drought treatment (14-days without watering) on *Mop1* WT, *mop1-1*, and B73 plants. Initially, all plants were watered normally and showed no significant differences in growth rate until reaching the V6 stage (Nielsen, 2019) when drought treatment was applied to randomly selected individuals from each group (Figure 5). We controlled for growth rate differences between individuals by beginning the drought treatment when an individual reached the V6 stage (auricle exposed). The growth rate was significantly delayed among all drought-treated plants compared to normally-watered controls, with *mop1-1* drought-treated plants taking the longest time to reach the V7 stage (Figure 5). After 14 days of drought treatment, water (7.5 L) was given to each plant and plants were normally watered throughout the duration of the experiment. While B73 and *Mop1* drought-treated plants recovered rapidly and approached the growth rate curve of normally-watered controls, *mop1-1* drought-treated plants significantly lagged behind, with several plants failing to reach reproductive competency (Figure 5A). Normally-watered *mop1-1* plants showed no significant differences in growth rates compared to normally-watered B73 and *Mop1* suggesting that MOP1 is required to recover from drought stress.

To determine the effects loss of MOP1 during drought had on reproductive development, we made observations for plant height at maturity, internodal length, ear emergence, number of ears, effective tassel branches, and the anthesis-silking interval (ASI) (Figure 5B; Supplementary Figure 4). Because stunted plant height is indicative of severe stress during vegetative development, we measured the average internodal length and the heights of plants at 90 days after sowing (DAS). Drought-treated plants were stunted and had reduced internodal lengths compared to normally-watered controls across genotypes (Figure 5B; Supplementary Figure 4). Reproductive development can also be affected by drought-stress and the magnitude of the effect is in some cases dependent on the stage of development in which the plant endures the stress. Because the drought-treatment in our study begins at the V6 stage, which is prior to the transition to reproductive development, we were able to determine the effects of vegetative stress on reproductive traits. To determine how drought affects tassel development, we characterized the effective tassel branches for each individual by measuring the ratio of tassel branches with functional anthers (i.e., anthers shedding pollen) to total tassel branches and found a drought-dependent decrease in effective tassel branches across genotypes, however, these differences were not significant (Supplementary Figure 4). The number of days until ear emergence and the number of ears per plant were also measured. It was found that drought treatment led to a significant delay in ear emergence and that *mop1-1* drought treated plants, but not B73 or *Mop1*, displayed a significant reduction in the number of ears per plant (Figure 5B; Supplementary Figure 4). In addition, *mop1-1* drought-treated plants displayed a significantly larger anthesis-silking interval compared to B73 and *Mop1*, suggesting that impaired recovery from drought stress in plants defective in MOP1 function has an effect on reproductive development and competency (Figure 5B).

## Discussion

Understanding the molecular mechanisms that contribute to plant responses to changing environments is essential to ensure that we can develop climate resistant plants that meet the increasing global demands on crop yield. RdDM and ABA-signaling are two critical gene-regulatory pathways that each influence how plants respond to environmental cues at specific developmental stages. The extent of the synergy between these two regulatory systems is largely uncharacterized in agronomically important crop plants, such as maize. To address this gap in knowledge, we recently conducted a transcriptomic analysis which demonstrated that loss of RdDM activity renders maize seedlings more susceptible to transcriptional changes as a result of ABA treatment, and that many genes were responsive to disruption of both regulatory networks after 8 h of phytohormone treatment (Vendramin et al., 2020). The differential response of the RdDM-deficient mutant to treatment with ABA and to water deprivation suggest that stressful growing conditions or exogenous of application of growth hormones like ABA might be sufficient stimuli to alter the epigenome of maize, and could be useful in crop epi-breeding platforms, which may enhance modern breeding efforts (Dalakouras and Vlachostergios, 2021). While this study identified and established synergy between these two networks in maize, interpretation of the results was confounded by the hierarchical nature of cascading transcriptional outcomes for both regulatory pathways, each dependent on varied *cis* and *trans*-regulatory elements.

Using an approach based on a temporal response to phytohormone treatment, we have identified immediate and direct MOP1-dependent transcriptional responses to ABA (MIMs) that are predicted to function upstream of genes responsive to longer periods of exposure to abiotic stress stimuli (MINs). These relatively few MIM genes, identified as unique 1 h Group C genes having homology with MOP1-dependent siRNAs, appear to be specific in their biological function. Using a GRN, we were able to establish a hierarchial relationship between predicted MIMs and MINs, where the MIMs identified in this study are predicted to regulate ~50% of genes differentially expressed after longer exposure to abiotic stress (8 h), suggesting a substantial impact on transcriptional responses by MIMs. The lack of multiple enriched GO terms associated with the 76 down-regulated MIMs is indicative of either a lack of biological specificity of these genes or a reflection of the complexity of regulation of genes in this category that may also be targets of an active demethylaion mechanisms by DNA glycosylases. For this study, only a subsets of possible regulatory features associated with RdDM activity were used to identify MIMs, and yet the predicted regulatory impact of the identified MIMs account for almost half of the MINs, suggesting that this may in fact be an underestimation of the contribution of MOP1 in establishing responsive transcriptional profiles. Additional analysis to include other RdDM regulatory features, such as proximity to specific categories of transposable elements (TEs) (Madzima et al., 2014; Vendramin et al., 2020) and contexts of cytosine methylation that establish boundaries between the TEs and adjacent protein-coding genes in maize (Gent et al., 2014; Li et al., 2015) might identify additional specific ABA-induced MIM genes. It is likely that an extensive genome-wide analysis will need to be pursued to elucidate specific examples of direct correlation between DNA methylation, chromatin marks and differential expression in these conditions, because prior work has demonstrated that these coordinated responses are hierarchical and inter-related, and often do not involve simple relationships between differential expression and hallmarks of RdDM (Madzima et al., 2014; Vendramin et al., 2020). Thus, a locus-specific approach was not attempted in this study.

There is already compelling evidence indicating that RdDM activity in maize has consequential effects on plant growth and development, affecting the male and female inflorescences (Dorweiler et al., 2000; Hultquist and Dorweiler, 2008) and ultimately seed yield (Barber et al., 2012). The study described herein, reveals that, consistent with *mop1-1* plants misexpressing genes involved in development (Vendramin et al., 2020), plants defective for RdDM are compromised in their growth rate recovery after water stress. This observation links the differences in transcriptional responses of maize *mop1-1* plants to differing abilities to recover from abiotic environmental influences, and highlights the physiological relevance of the gene expression phenotypes of RdDM-deficient plants.

Collectively, this data suggests that MOP1 activity is required for preparedness to respond, early response and later response to ABA signaling at the level of gene expression, and may indicate that MOP1, a component of RdDM in maize, functions in plant response to stressful growth conditions. Future work will include molecular characterization of the MIMs to identify the architecture of upstream *cis*-regulatory elements of these genes.

## Data Availability Statement

The datasets presented in this study can be found in online repositories. RNA-sequencing data is available at the National Center for Biotechnology Information (NCBI) Gene Expression Omnibus (Edgar et al., 2002) through GEO Series accession number GSE179629.

## Author Contributions

TM, SV, JL, and KM designed the research. SV performed the seedling ABA-induction experiments and RNA-seq library preparation. JL and PL performed the drought stress experiments. TM and KM performed analysis and interpretation of seedling ABA RNA-seq data. KL assisted with bioinformatic analysis and interpretation. TM and KM wrote the manuscript. All authors edited and/or reviewed the original manuscript, and contributed to the article and approved the submitted version.

## Conflict of Interest

The authors declare that the research was conducted in the absence of any commercial or financial relationships that could be construed as a potential conflict of interest.

## Publisher's Note

All claims expressed in this article are solely those of the authors and do not necessarily represent those of their affiliated organizations, or those of the publisher, the editors and the reviewers. Any product that may be evaluated in this article, or claim that may be made by its manufacturer, is not guaranteed or endorsed by the publisher.

## Supplementary Material

Supplementary Figure 1 Threshold normalization for uniquely mapping reads. The mapped reads from RNA-seq after 1 h (this study; Illumina NovaSeq platform) and 8 h (Vendramin et al., 2020; Illumina HiSeq platform) of ABA induction were simultaneously, bioinformatically processed and mapped to the B73 reference genome (AGP B73v4) (Jiao et al., 2017). Read quality score thresholds were normalized using HISAT2 (“–score-min”) between sequencing platforms. Based on consistency between replicates as well as differences in distributions of mapping qualities between sequencing platforms, the HISAT2 “–score-min” parameter was chosen to normalize the number of uniquely mapped reads across datasets. HISAT2 filters reads based on a threshold were defined by the slope a linear function between mapping quality score and read length. HISAT2 slope filter threshold for 1 h = −0.2 and for 8 h = −0.6. Genotype-treatment samples include wild type MS (wm), wild type ABA (wa), mutant MS (mm), mutant ABA (ma).

Supplementary Figure 2 Re-analysis of differentially expressed genes (DEGs) after 8h ABA treatment. **(A)** Venn diagram of overlap in the identity of DEGs (log2 FC ≥ 0.95, FDR < 0.05) after 8 h treatment with ABA compared to control in homozygous mutant (a_up and a_down) and wild type plants (b_up and b_down). **(B)** Venn diagram of overlap in the identity of DEGs (log2 FC ≥ 0.95, FDR < 0.05) in homozygous mutant compared to wild type plants after 8 h treatment with ABA (c_up and c_down) or 1 h treatment with MS (d_up and d_down).

Supplementary Figure 3 Analysis of differentially expressed genes (DEGs) in 8h/1h comparisons. Venn diagram of overlap in the identity DEGs (log2 FC ≥ 0.95, FDR < 0.05) in each analysis 8 h/1 h (Table 4) were compared. **(A)** identity of analysis groups E and F DEGs. **(B)** identity of analysis groups G and H. **(C)** identity of analysis groups E and G. **(D)** identity of analysis groups F and H.

Supplementary Figure 4 Additional physiological observations from drought stress experiment. Graphs show **(A)** average plant height, **(B)** effective tassel branches, and **(C)** ear emergence in number of days after sowing (DAS) in normally watered plants or after water was withheld for 14 days (Drought treated). Data is shown for B73 inbred lines under different watering conditions, and wild type (*Mop1*) and homozygous *mop1-1* individuals segregating within a family. Significant differences (*p* < 0.05) between groups are indicated by an asterisk (^*^) or no significance (NS).

Supplementary Table 1 List of DEGs for mop1 ABA-treated samples.

Supplementary Table 2 List of DEGs for 1 h, 8 h and 8 h/1 h Venn diagrams.

Supplementary Table 3 List of 1 h Group C 97 and 76 genes and GRN targets.

Supplementary Table 4 List of secondary targets.

## References

[B1] Barber W. T. Zhang W. Win H. Varala K. K. Dorweiler J. E. Hudson M. E.. (2012). Repeat associated small RNAs vary among parents and following hybridization in maize. Proc.Natl. Acad. Sci. U. S. A. 109, 10444–10449. 10.1073/pnas.1202073109 22689990 PMC3387101

[B2] Bolanos J. Edmeades G. O. (1996). The importance of the anthesis-silking interval in breeding for drought tolerance in tropical maize. Field Crops Res. 48, 65–80. 10.1016/0378-4290(96)00036-6

[B3] Chang Y. N. Zhu C. Jiang J. Zhang H. Zhu J. K. Duan C. G. (2020). Epigenetic regulation in plant abiotic stress responses. J. Integr. Plant Biol. 62, 563–580. 10.1111/jipb.12901 31872527

[B4] Chen S. Zhou Y. Chen Y. Gu J. (2018). fastp: an ultra-fast all-in-one FASTQ preprocessor. Bioinformatics 34, i884–i890. 10.1093/bioinformatics/bty560 30423086 PMC6129281

[B5] Chen Y. Lun A. T. Smyth G. K. (2016). From reads to genes to pathways: differential expression analysis of RNA-Seq experiments using Rsubread and the edgeR quasi-likelihood pipeline. F1000Res. 5:1438. 10.12688/f1000research.8987.2 27508061 PMC4934518

[B6] Claassen M. M. Shaw R. H. (1970). Water deficit effects on corn. II. Grain components1. Agron. J. 62, 652–655.

[B7] Dalakouras A. Vlachostergios D. (2021). Epigenetic approaches for crop breeding: current status and perspectives. J. Exp. Bot. erab227. 10.1093/jxb/erab227 34017985

[B8] Dorweiler J. E. Carey C. C. Kubo K. M. Hollick J. B. Kermicle J. L. Chandler V. L. (2000). Mediator of paramutation1 is required for establishment and maintenance of paramutation at multiple maize loci. Plant Cell 12, 2101–2118. 10.1105/tpc.12.11.2101 11090212 PMC150161

[B9] Edgar R. Domrachev M. Lash A. E. (2002). Gene expression omnibus: NCBI gene expression and hybridization array data repository. Nucleic Acids Res. 30, 207–210. 10.1093/nar/30.1.207 11752295 PMC99122

[B10] FAO: Food and Agriculture Organization of the United Nations (2017). FAOSTAT Statistical Database. Rome: FAO.

[B11] Forestan C. Aiese Cigliano R. Farinati S. Lunardon A. Sanseverino W. Varotto S. (2016). Stress-induced and epigenetic-mediated maize transcriptome regulation study by means of transcriptome reannotation and differential expression analysis. Sci. Rep. 6:30446. 10.1038/srep30446 27461139 PMC4962059

[B12] Forestan C. Farinati S. Aiese Cigliano R. Lunardon A. Sanseverino W. Varotto S. (2017). Maize RNA PolIV affects the expression of genes with nearby TE insertions and has a genome-wide repressive impact on transcription. BMC Plant Biol. 17:161. 10.1186/s12870-017-1108-1 29025411 PMC5639751

[B13] Forestan C. Farinati S. Zambelli F. Pavesi G. Rossi V. Varotto S. (2020). Epigenetic signatures of stress adaptation and flowering regulation in response to extended drought and recovery in Zea mays. Plant Cell Environ. 43, 55–75. 10.1111/pce.13660 31677283

[B14] Gent J. I. Madzima T. F. Bader R. Kent M. R. Zhang X. Stam M.. (2014). Accessible DNA and relative depletion of H3K9me2 at maize loci undergoing RNA-directed DNA methylation. Plant Cell 26, 4903–4917. 10.1105/tpc.114.130427 25465407 PMC4311197

[B15] Gupta A. Rico-Medina A. Cano-Delgado A. I. (2020). The physiology of plant responses to drought. Science 368, 266–269. 10.1126/science.aaz7614 32299946

[B16] Heberle E. Bardet A. F. (2019). Sensitivity of transcription factors to DNA methylation. Essays Biochem. 63, 727–741. 10.1042/EBC20190033 31755929 PMC6923324

[B17] Huang J. Zheng J. Yuan H. McGinnis K. (2018). Distinct tissue-specific transcriptional regulation revealed by gene regulatory networks in maize. BMC Plant Biol. 18:111. 10.1186/s12870-018-1329-y 29879919 PMC6040155

[B18] Hultquist J. F. Dorweiler J. E. (2008). Feminized tassels of maize mop1 and ts1 mutants exhibit altered levels of miR156 and specific SBP-box genes. Planta 229, 99–113. 10.1007/s00425-008-0813-2 18800226

[B19] Jiao Y. Peluso P. Shi J. Liang T. Stitzer M. C. Wang B.. (2017). Improved maize reference genome with single-molecule technologies. Nature 546, 524–527. 10.1038/nature22971 28605751 PMC7052699

[B20] Kim J. M. Sasaki T. Ueda M. Sako K. Seki M. (2015). Chromatin changes in response to drought, salinity, heat, and cold stresses in plants. Front. Plant Sci. 6:114. 10.3389/fpls.2015.00114 25784920 PMC4345800

[B21] Koster J. Rahmann S. (2018). Snakemake-a scalable bioinformatics workflow engine. Bioinformatics 34:3600. 10.1093/bioinformatics/bty350 29788404

[B22] Kurtzer G. M. Sochat V. Bauer M. W. (2017). Singularity: scientific containers for mobility of compute. PLoS ONE 12:e0177459. 10.1371/journal.pone.0177459 28494014 PMC5426675

[B23] Li Q. Gent J. I. Zynda G. Song J. Makarevitch I. Hirsch C. D.. (2015). RNA-directed DNA methylation enforces boundaries between heterochromatin and euchromatin in the maize genome. Proc. Natl. Acad Sci U. S. A. 112, 14728–14733. 10.1073/pnas.1514680112 26553984 PMC4664327

[B24] Love M. I. Huber W. Anders S. (2014). Moderated estimation of fold change and dispersion for RNA-seq data with DESeq2. Genome Biol. 15:550. 10.1186/s13059-014-0550-8 25516281 PMC4302049

[B25] Lu F. Wang K. Yan L. Peng Y. Qu J. Wu J.. (2020). Isolation and characterization of maize ZmPP2C26 gene promoter in drought-response. Physiol. Mol. Biol. Plants 26, 2189–2197. 10.1007/s12298-020-00910-2 33268922 PMC7688808

[B26] Ma Y. Cao J. He J. Chen Q. Li X. Yang Y. (2018). Molecular mechanism for the regulation of ABA homeostasis during plant development and stress responses. Int. J. Mol. Sci. 19:3643. 10.3390/ijms19113643 30463231 PMC6274696

[B27] Madzima T. F. Huang J. McGinnis K. M. (2014). Chromatin structure and gene expression changes associated with loss of MOP1 activity in Zea mays. Epigenetics 9, 1047–1059. 10.4161/epi.29022 24786611 PMC4143406

[B28] Martin M. (2011). Cutadapt removes adapter sequences from high-throughput sequencing reads. EMBnet J. 17, 10–12. 10.14806/ej.17.1.200

[B29] Nielsen R. (2019). Grain Fill Stages in Corn. Available online at: www.agry.purdue.edu/ext/corn/news/timeless/grainfill.html

[B30] Pertea M. Kim D. Pertea G. M. Leek J. T. Salzberg S. L. (2016). Transcript-level expression analysis of RNA-seq experiments with HISAT, StringTie and Ballgown. Nat. Protoc. 11, 1650–1667. 10.1038/nprot.2016.095 27560171 PMC5032908

[B31] Qin F. Sakuma Y. Li J. Liu Q. Li Y. Q. Shinozaki K.. (2004). Cloning and functional analysis of a novel DREB1/CBF transcription factor involved in cold-responsive gene expression in *Zea mays* L. Plant Cell Physiol. 45, 1042–1052. 10.1093/pcp/pch118 15356330

[B32] Sallam N. Moussa M. (2021). DNA methylation changes stimulated by drought stress in ABA-deficient maize mutant vp10. Plant Physiol. Biochem. 160, 218–224. 10.1016/j.plaphy.2021.01.024 33515971

[B33] Song L. Huang S.-s. C. Wise A. Castanon R. Nery J. R. Chen H.. (2016). A transcription factor hierarchy defines an environmental stress response network. Science 354:aag1550. 10.1126/science.aag1550 27811239 PMC5217750

[B34] Song N. Xu Z. Wang J. Qin Q. Jiang H. Si W.. (2018). Genome-wide analysis of maize CONSTANS-LIKE gene family and expression profiling under light/dark and abscisic acid treatment. Gene 673, 1–11. 10.1016/j.gene.2018.06.032 29908279

[B35] Takahashi F. Kuromori T. Sato H. Shinozaki K. (2018). Regulatory Gene Networks in Drought Stress Responses and Resistance in Plants. Adv. Exp. Med. Biol. 1081, 189–214. 10.1007/978-981-13-1244-1_11 30288711

[B36] Takahashi F. Kuromori T. Urano K. Yamaguchi-Shinozaki K. Shinozaki K. (2020). Drought Stress Responses and Resistance in Plants: From Cellular Responses to Long-Distance Intercellular Communication. Front. Plant Sci. 11:556972. 10.3389/fpls.2020.556972 33013974 PMC7511591

[B37] Tian T. Liu Y. Yan H. You Q. Yi X. Du Z.. (2017). agriGO v2.0: a GO analysis toolkit for the agricultural community, 2017 update. Nucleic Acids Res. 45, W122–W129. 10.1093/nar/gkx382 28472432 PMC5793732

[B38] Van den Broeck L. Dubois M. Vermeersch M. Storme V. Matsui M. Inze D. (2017). From network to phenotype: the dynamic wiring of an Arabidopsis transcriptional network induced by osmotic stress. Mol. Syst. Biol. 13:961. 10.15252/msb.20177840 29269383 PMC5740496

[B39] Vendramin S. Huang J. Crisp P. A. Madzima T. F. McGinnis K. M. (2020). Epigenetic regulation of ABA-induced transcriptional responses in maize. G3 (Bethesda). 10, 1727–1743. 10.1534/g3.119.400993 32179621 PMC7202028

[B40] Wang L. Qiao H. (2020). Chromatin regulation in plant hormone and plant stress responses. Curr. Opin. Plant Biol. 57, 164–170. 10.1016/j.pbi.2020.08.007 33142261 PMC8237520

[B41] Xiang Y. Sun X. Gao S. Qin F. Dai M. (2017). Deletion of an endoplasmic reticulum stress response element in a ZmPP2C-A gene facilitates drought tolerance of maize seedlings. Mol. Plant 10, 456–469. 10.1016/j.molp.2016.10.003 27746300

[B42] Yilmaz A. Nishiyama M. Y. Fuentes B. G. Jr. Souza G. M. Janies D. Gray J.. (2009). GRASSIUS: a platform for comparative regulatory genomics across the grasses. Plant Physiol. 149, 171–180. 10.1104/pp.108.128579 18987217 PMC2613736

