# Supplementary material for: Direct and Indirect Transcriptional Effects of Abiotic Stress in Zea mays Plants Defective in RNA-Directed DNA Methylation
Source: Front Plant Sci. 2021 Aug 19;12:694289. doi: 10.3389/fpls.2021.694289 (PMC8418275; doi:10.3389/fpls.2021.694289)
Supplement: Supplementary Figure 1 — Threshold normalization for uniquely mapping reads. The mapped reads from RNA-seq after 1 h (this study; Illumina NovaSeq platform) and 8 h (Vendramin et al., 2020; Illumina HiSeq platform) of ABA induction were simultaneously, bioinformatically processed and mapped to the B73 reference genome (AGP B73v4) (Jiao et al., 2017). Read quality score thresholds were normalized using HISAT2 (“–score-min”) between sequencing platforms. Based on consistency between replicates as well as differences in distributions of mapping qualities between sequencing platforms, the HISAT2 “–score-min” parameter was chosen to normalize the number of uniquely mapped reads across datasets. HISAT2 filters reads based on a threshold were defined by the slope a linear function between mapping quality score and read length. HISAT2 slope filter threshold for 1 h = −0.2 and for 8 h = −0.6. Genotype-treatment samples include wild type MS (wm), wild type ABA (wa), mutant MS (mm), mutant ABA (ma). [file Presentation_1.PPTX]

## Slide 1
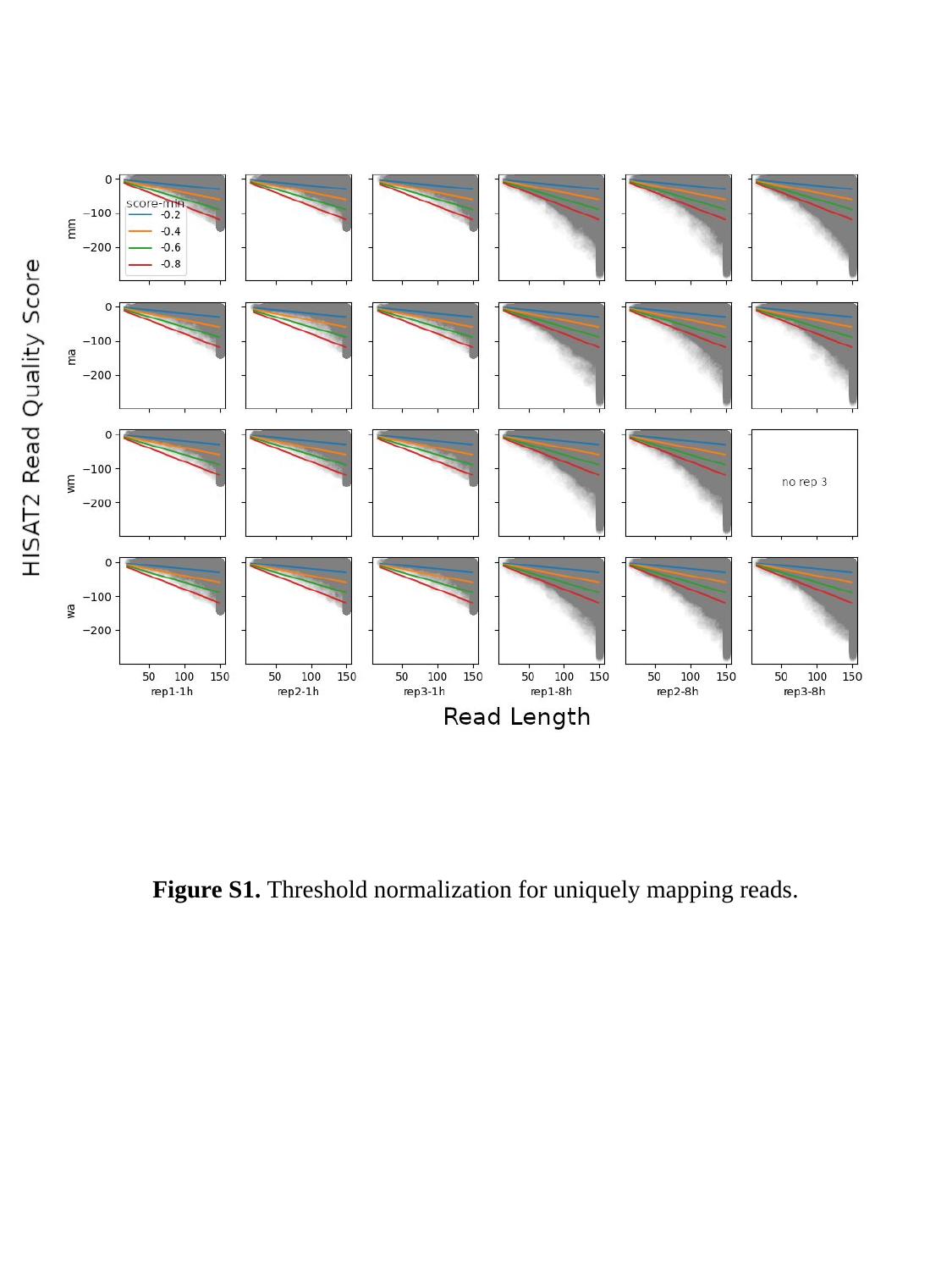

Figure S1. Threshold normalization for uniquely mapping reads.

## Slide 2
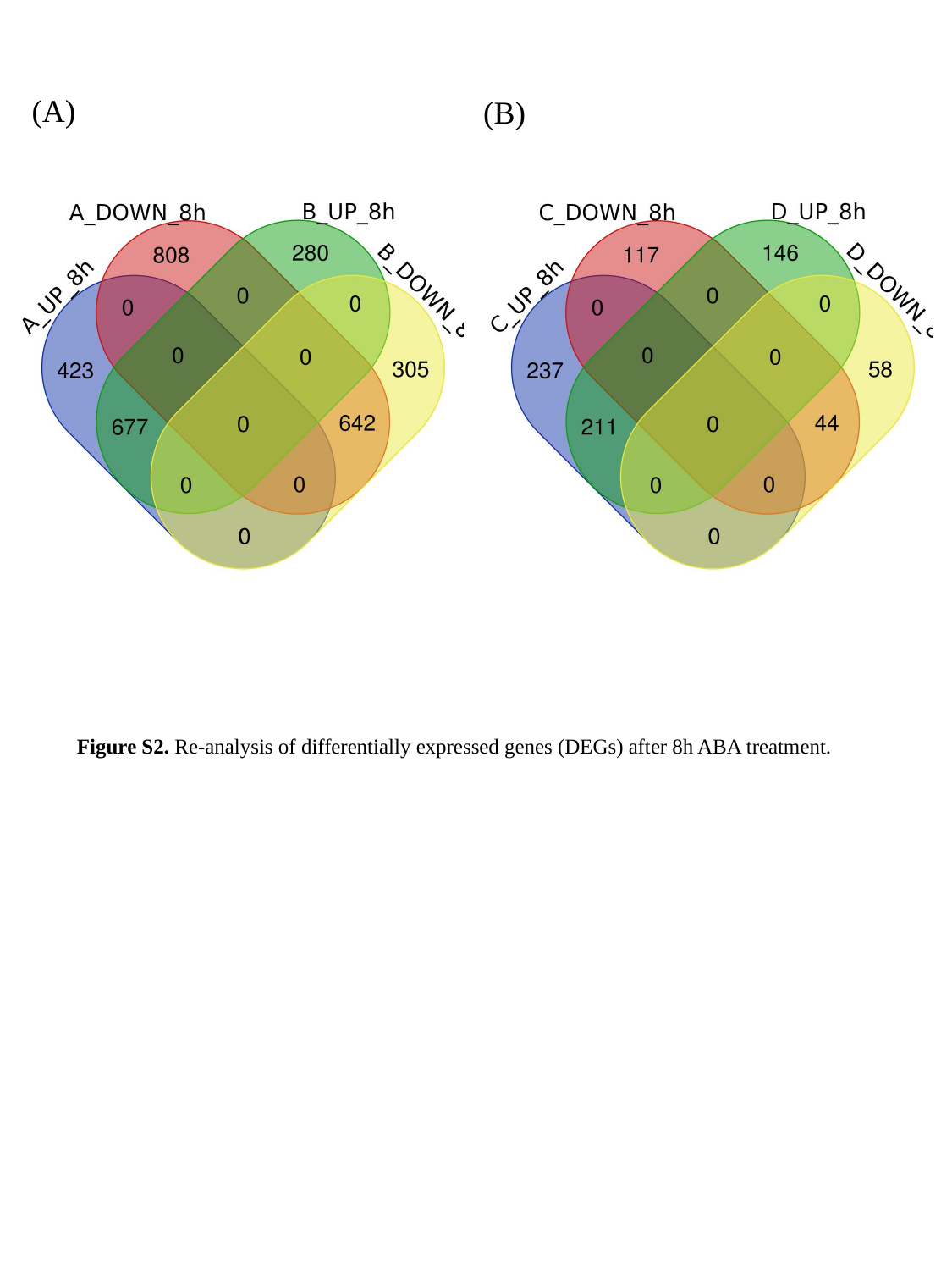

(A)
(B)
Figure S2. Re-analysis of differentially expressed genes (DEGs) after 8h ABA treatment.

## Slide 3
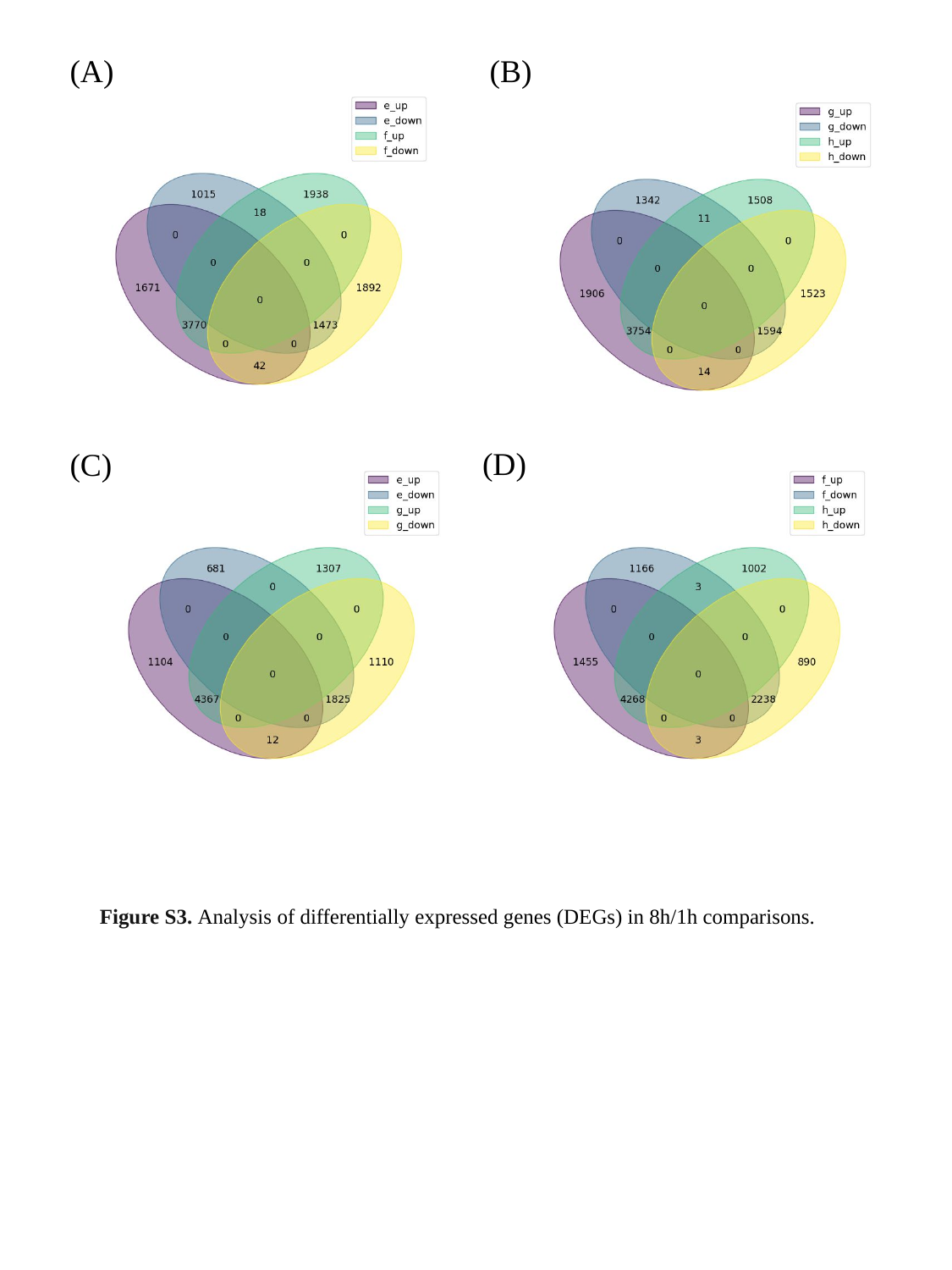

(A)
(B)
(D)
(C)
Figure S3. Analysis of differentially expressed genes (DEGs) in 8h/1h comparisons.

## Slide 4
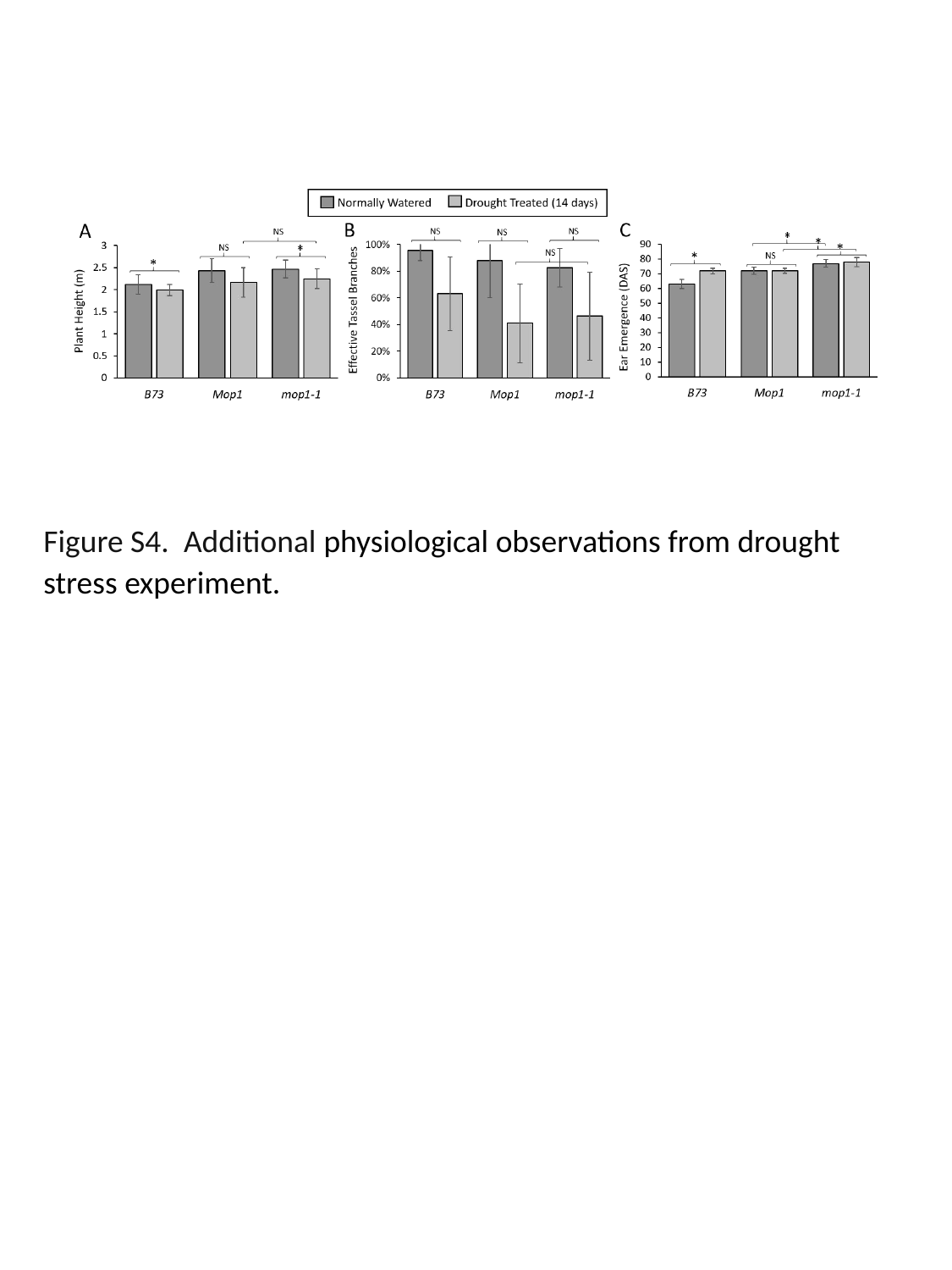

Figure S4. Additional physiological observations from drought stress experiment.
