# Supplementary figures and images for: Direct and Indirect Transcriptional Effects of Abiotic Stress in Zea mays Plants Defective in RNA-Directed DNA Methylation
Source: Front Plant Sci. 2021 Aug 19;12:694289. doi: 10.3389/fpls.2021.694289 (PMC8418275; doi:10.3389/fpls.2021.694289)

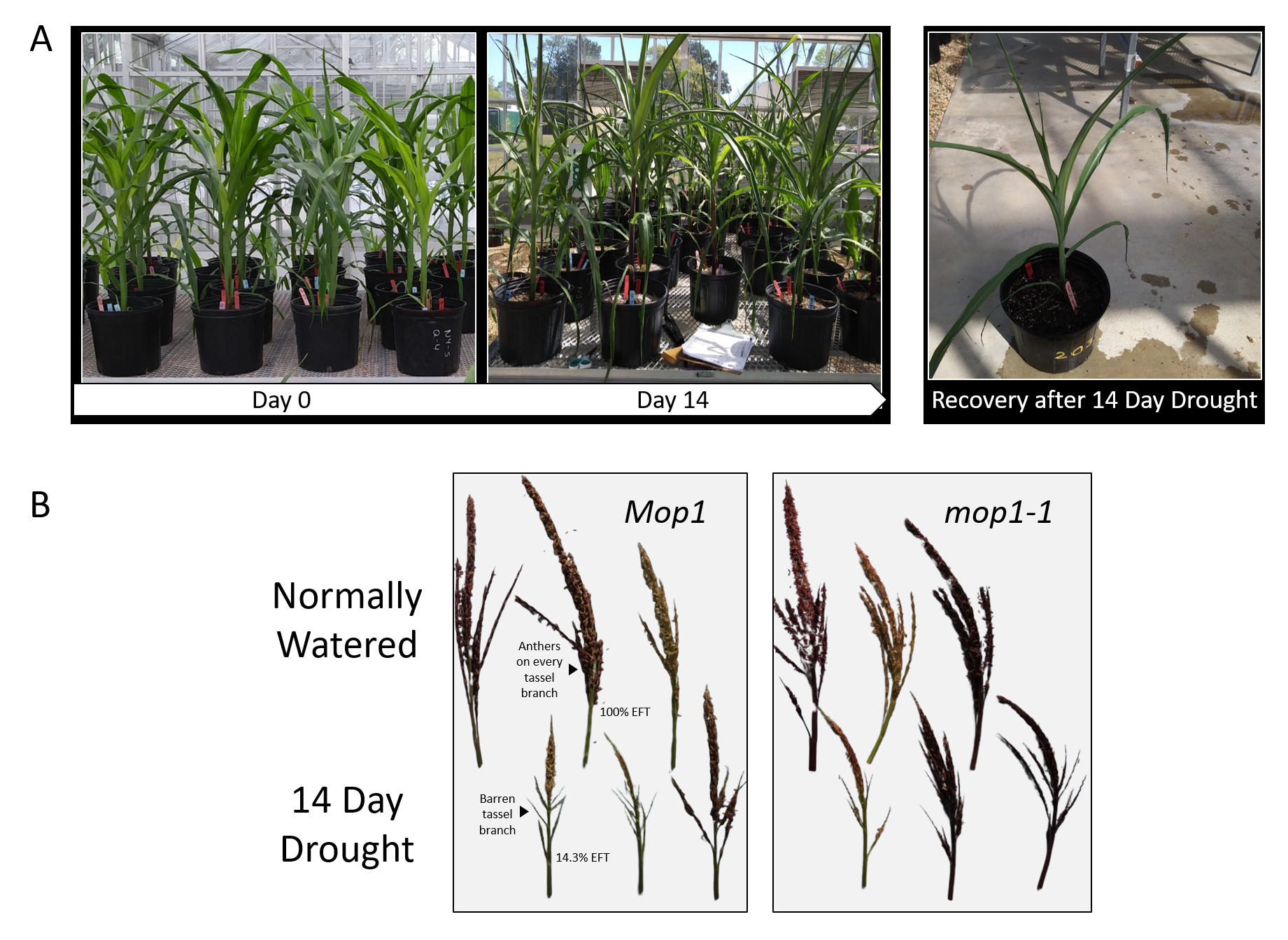

Supplement: Supplementary file 6 [file Image_1.PNG]
